# Supplementary figures and images for: Predicting clinical outcomes of SARS-CoV-2 infection during the Omicron wave using machine learning
Source: PLoS One. 2024 Apr 25;19(4):e0290221. doi: 10.1371/journal.pone.0290221 (PMC11045098; doi:10.1371/journal.pone.0290221)

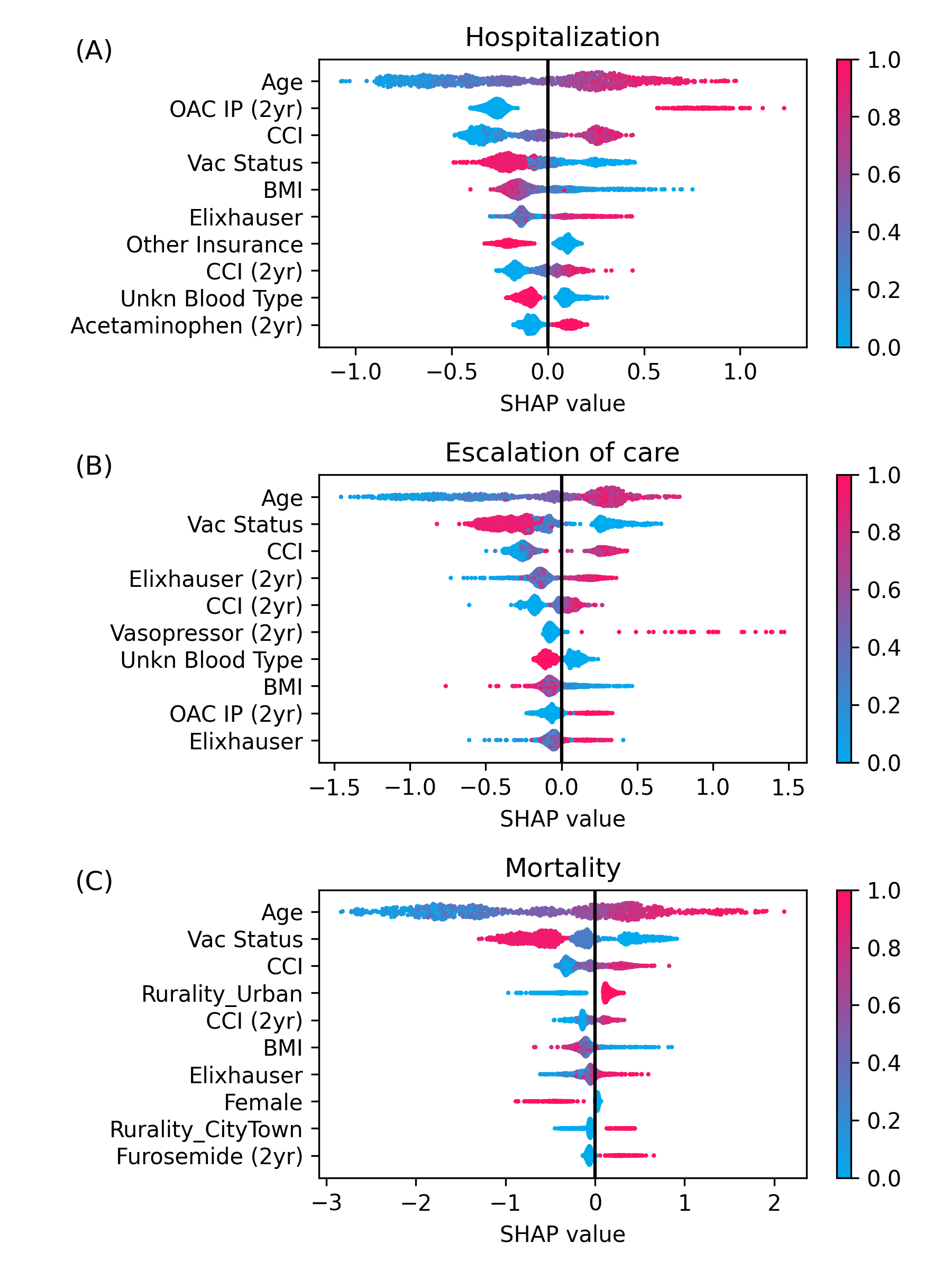

Supplement: S1 Fig — (A) hospitalization, (B) escalation of care, and (C) mortality. Covariates are listed in order of highest to lowest impact (based on absolute mean SHAP value) along the y-axis. Each blue or red point represents a patient’s specified covariate value; that value is color coded in a heat map fashion per the legend. The x-axis is the SHAP value for the specific covariate, with SHAP values greater than 0 indicating higher predicted risk contribution and values less than 0 indicating lower predicted risk contribution for the given outcome. (TIF) [file pone.0290221.s003.tif]

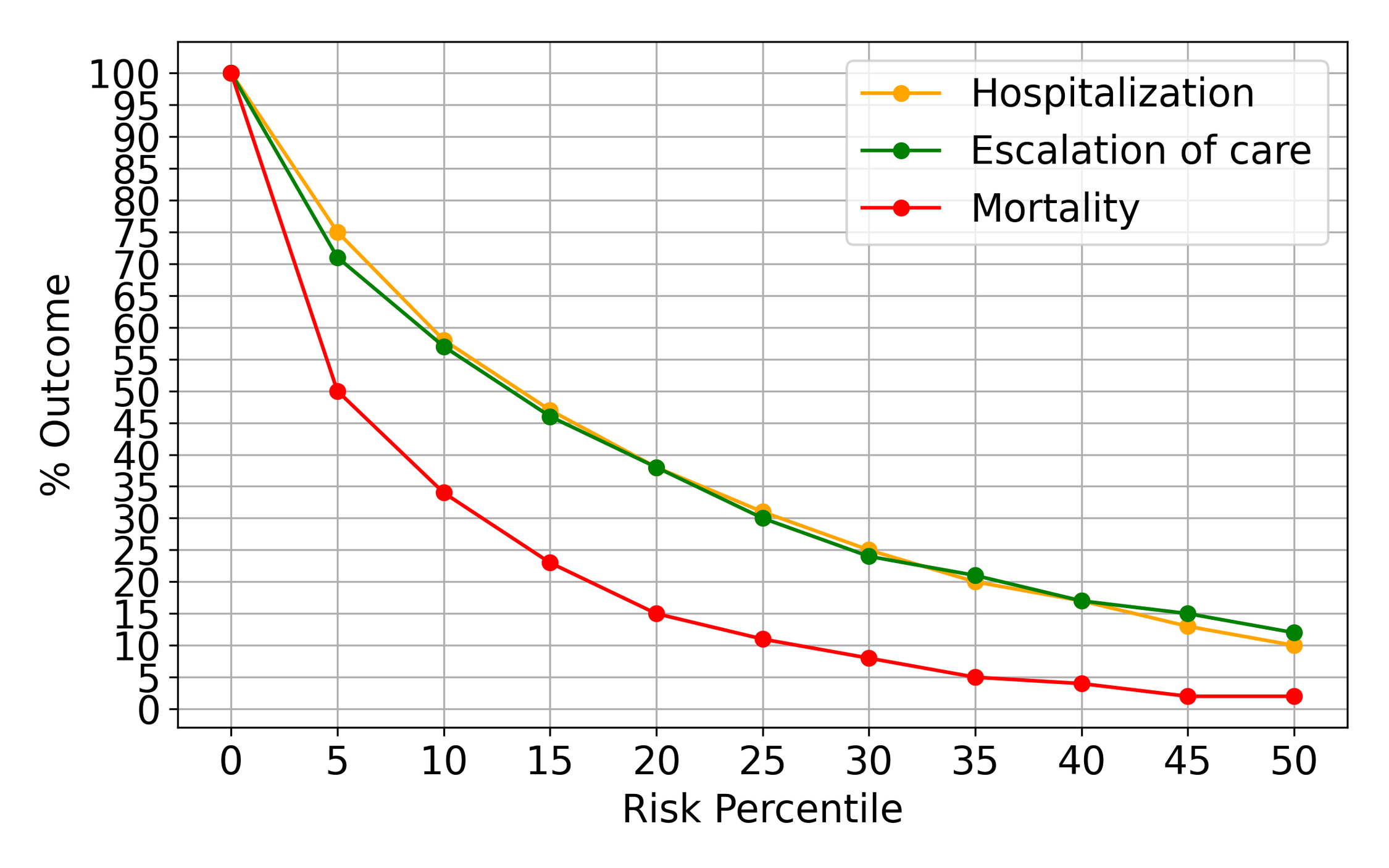

Supplement: S2 Fig — A plot of the percentile of a target outcome as it relates to the risk percentile for our test population. (TIF) [file pone.0290221.s004.tif]
